# Adenosine Pathway Activation Defines Genetically Linked Immunosuppressive Subtypes in Solid Tumor Brain Metastases

**DOI:** 10.3390/cancers18071087

**Published:** 2026-03-26

**Authors:** Arthur Bauer, Annette Arndt, Luisa Reichenbach, Niklas Gebauer, Matthias Müller, Christian Neumann, Chris Schulz, Konrad Steinestel, Raban Heller, Emil Chteinberg, Hauke Busch, Armin Riecke, Axel Künstner, Hanno Witte

**Affiliations:** 1 Department of Hematology and Oncology, Bundeswehrkrankenhaus Ulm, Oberer Eselsberg 40, 89081 Ulm, Germanymatthias27mueller@bundeswehr.org (M.M.);; 2 Institute for Pathology und Molecularpathology, Bundeswehrkrankenhaus Ulm, Oberer Eselsberg 40, 89081 Ulm, Germany; annette2arndt@bundeswehr.org (A.A.);; 3 Department for Hematology and Oncology, University Hospital Schleswig-Holstein Campus Lübeck, Ratzeburger Allee 160, 23538 Lübeck, Germany; niklas.gebauer@uksh.de; 4 University Cancer Center Schleswig-Holstein (UCCSH), UKSH Campus Lübeck, Ratzeburger Allee 160, 23538 Lübeck, Germany; hauke.busch@uni-luebeck.de (H.B.); axel.kuenstner@uni-luebeck.de (A.K.); 5 Department of Neurosurgery, Bundeswehrkrankenhaus Ulm, Oberer Eselsberg 40, 89081 Ulm, Germany; chris.schulz@uni-ulm.de; 6 Central Clinical Management, Bundeswehrkrankenhaus Ulm, Oberer Eselsberg 40, 89081 Ulm, Germany; raban.heller@uni-ulm.de; 7 Institute for Pathology, University Ulm, Albert Einstein Allee 23, 89081 Ulm, Germany; emil.chteinberg@uni-ulm.de; 8 Medical Systems Biology Group, Lübeck Institute of Experimental Dermatology, University of Lübeck, Ratzeburger Allee 160, 23538 Lübeck, Germany

**Keywords:** brain metastasis, adenosine pathway, CD39, CD73, immunotherapy

## Abstract

**Introduction:** Brain metastases represent a major clinical challenge due to a distinct immunosuppressive microenvironment and limited, heterogeneous efficacy of PD-1/PD-L1 immune checkpoint inhibition. The adenosine pathway, mediated by the ectonucleotidases CD39 and CD73, has emerged as an alternative immune escape mechanism, yet its relevance in brain metastases across tumor entities remains insufficiently characterized. **Methods:** We conducted targeted panel sequencing of brain metastases from multiple primary tumor entities and evaluated compartment-resolved expression of CD39, CD73, and PD-L1 by immunohistochemistry, distinguishing tumor cell and immune cell expression. Tumor mutational burden (TMB), recurrent gene alterations, and gene fusions were analyzed and integrated with immune marker profiles to define immunogenomic subtypes. **Results:** Brain metastases displayed a heterogeneous mutational landscape with recurrent alterations including *TP53*, *KRAS*, *PIK3CA*, and *APC*. CD39 and CD73 expression was frequent and highly variable, occurring on both tumor cells and tumor-infiltrating immune cells, and only partially overlapping with PD-L1 expression. A substantial subset of cases exhibited an adenosine-high phenotype despite low or absent PD-L1. Marker-associated enrichment analyses identified distinct genetic correlates, including enrichment of *KRAS* alterations in tumors with CD39/CD73 positivity on malignant cells, and APC/PIK3CA-associated patterns linked to immune compartment marker expression. TMB did not significantly differ across major tumor entity groups. Gene fusions were detected in a subset of tumors but were largely independent of immune phenotypes. **Conclusions:** Adenosine pathway activation is a frequent, genetically associated immune escape feature of brain metastases that complements PD-L1-based stratification. Integrating CD39/CD73 with PD-L1 enables actionable immunogenomic subtyping and supports rational immunotherapy strategies targeting adenosine-mediated immunosuppression.

## Simple Summary

Brain metastases represent a major challenge in cancer treatment because tumors in the brain often evade immune responses and respond inconsistently to immunotherapy. While many current immunotherapy strategies focus on the PD-1/PD-L1 pathway, other immune escape mechanisms may also play an important role. In this study, we analyzed the genetic landscape and immune marker expression in brain metastases from different primary tumors. We found that the adenosine pathway, mediated by the enzymes CD39 and CD73, is frequently active in brain metastases and often occurs independently of PD-L1 expression. Importantly, certain genetic alterations, including KRAS, APC, and PIK3CA mutations, were associated with specific immune phenotypes. By integrating genomic data with immune marker profiles, we identified distinct immunogenomic subtypes of brain metastases. These findings suggest that targeting the adenosine pathway could complement existing immunotherapy approaches and may help improve treatment strategies for patients with brain metastases.

## 1. Introduction

Recent advances in cancer immunotherapy and molecularly stratified treatment concepts have substantially improved outcomes across multiple solid tumor entities, even in advanced disease stages [1,2,3]. In parallel, precision oncology has become an integral component of contemporary clinical management, enabling biomarker-driven therapeutic decisions increasingly implemented within molecular tumor boards [4].

Brain metastases (BMs) remain a major clinical challenge across solid malignancies and are associated with limited therapeutic options and poor prognosis [5]. Standard-of-care treatment relies on surgical resection and stereotactic radiotherapy; however, effective systemic strategies with consistent intracranial activity remain scarce [6,7]. The central nervous system (CNS) represents a unique biological niche characterized by restricted drug delivery due to the blood–brain barrier (BBB) and a distinct immune microenvironment, which may promote immune evasion and therapeutic resistance [8]. Consequently, patients with BMs are frequently underrepresented in clinical trials, limiting evidence-based systemic treatment strategies [9]. A deeper mechanistic understanding of the molecular and immunological determinants shaping the CNS metastatic niche is therefore critical to guide novel therapeutic approaches [10].

Among emerging immunotherapeutic concepts, targeting the adenosine pathway has gained increasing attention as a strategy to counteract tumor-associated immunosuppression [11]. The ectonucleotidases CD39 (ENTPD1) and CD73 (NT5E) mediate extracellular ATP catabolism, leading to adenosine accumulation and suppression of anti-tumor immune responses [12,13]. Preclinical and early translational studies suggest that blockade of this pathway may restore immune effector function and synergize with immune checkpoint inhibition across tumor entities, positioning CD39 and CD73 as promising targets, particularly in immune-excluded or immunosuppressed settings [14,15,16,17].

Comprehensive molecular profiling has shown that BMs exhibit both shared and entity-specific genomic features compared with matched primary tumors, highlighting pronounced intertumoral heterogeneity [18]. Although recurrent alterations affecting cell cycle regulation, DNA damage response, and oncogenic signaling have been described across cancer types, the extent to which these programs converge on immune escape mechanisms remains insufficiently defined [19].

Here, we perform an integrated molecular and immune profiling of BMs derived from diverse solid tumor entities, with a particular focus on the adenosine axis as a candidate immunosuppressive checkpoint. By combining targeted sequencing with immunohistochemical assessment of CD39 and CD73, as well as established immune checkpoint biomarkers including PD-L1 TPS, CPS, and immune cell (IC) scoring, we delineate the genomic landscape of brain metastases and characterize tumor-intrinsic and microenvironmental immunoregulatory patterns. This integrative approach provides a framework for biomarker-driven stratification of CNS metastatic disease and supports the rational development of adenosine pathway-directed immunotherapeutic strategies.

## 2. Methods

### 2.1. Study Design and Patient Selection

This monocentric retrospective study included 49 patients diagnosed with BMs. Lung cancer was the predominant primary malignancy with 20 cases, followed by gastrointestinal (GI) cancer (*n* = 11), gynecological cancer (*n* = 9) and urologic cancer subtypes (*n* = 4), head and neck cancer (*n* = 3) and skin cancer (*n* = 2; see Supplementary Figure S1). Eligible cases were those treated at the center between 2014 and 2024, with patients providing written informed consent for the use of their clinical data and further pathological analysis of their sample material. The study was reviewed and approved by the Ethics Committee of the University of Ulm (Ref. No. 503-20). The cohort underwent comprehensive clinical analysis. Data were extracted from electronic patient files, including demographic characteristics, treatment details, treatment responses, and pathological diagnostic information.

### 2.2. Histopathological Evaluation

Biopsy specimens and archived slides pertinent to the current investigation were procured from the Institute of Pathology and Molecular Pathology at Bundeswehrkrankenhaus Ulm. Subsequently, a sequential process of histopathological reassessment and additional immunohistochemical analyses was systematically conducted.

### 2.3. Immunohistochemistry

Immunohistochemistry was performed to assess CD39 (HPA014067, Sigma Aldrich (St. Louis, MO, USA); 1:100) and CD73 (HPA017357, Sigma Aldrich; 1:500) expression on tumor and immune cells. Marker establishment included optimization steps as well as appropriate positive and negative controls. PD-L1 expression and scoring were evaluated as previously described. Following deparaffinization and rehydration, 4-μm sections underwent antigen retrieval using cell conditioning buffer 1 (CC1) and were incubated with primary antibodies, followed by biotinylated secondary antibodies. Immunoreactivity was visualized using the Ventana OptiView DAB detection system. Mean expression scores were calculated per case. Antibodies and positivity cut-offs are summarized in Supplementary Table S1. All stainings were performed according to the manufacturer’s instructions on a Ventana Benchmark Ultra immunostainer, using normal human tonsil tissue as a positive control [20].

### 2.4. CD39, CD73 and PD-L1 Scoring

Percentage expression levels of CD39 and CD73 have been evaluated in tumor cells and immune cells using conventional microscopy. Referring to PD-L1 scoring proposed by Schildhaus et al., the immune proportional score (IPS), defined as the percentage of immune cells showing any membrane expression of CD39+ or CD73+ (four subgroups: 0 ≤ 1%; 1 = 1–5%; 2 = 5–10%; 3 ≥ 10%), was calculated for each case. By analogy, the tumor proportional score (TPS) was defined as the percentage of viable tumor cells showing any membrane expression of CD39 or CD73 [21]. Additionally, we performed PD-L1 scoring (TPS and immune cell (IC) score) in all cases in concordance with Schildhaus et al. as previously described in salivary gland carcinomas [20].

### 2.5. Molecular Profiling

All analyses were performed on archived formalin-fixed and paraffin-embedded (FFPE) tissue samples. For sequencing analysis, DNA was isolated from tumor tissue using the Maxwell^®^ CSC DNA FFPE kit, operated on a Maxwell^®^ CSC instrument, following the manufacturer’s protocols (Promega, Madison, WI, USA).

The concentrations of the isolated nucleic acids were measured fluorometrically using the Qubit 4 fluorometer (Thermo Fisher Scientific, Waltham, MA, USA).

For DNA-based targeted next-generation sequencing (NGS), the TruSight Oncology 500 kit (San Diego, CA, USA) was utilized. Genomic DNA (100 ng) was fragmented by ultrasonication (ME 220 focused ultrasonicator, Brighton, UK). DNA-based library preparation was conducted precisely according to the manufacturer’s recommendations. The quantity and quality assessments, as well as the normalization and pooling of the prepared libraries, followed the TruSight Oncology 500 protocol (Supplementary Table S2). NGS was carried out by loading 1 pM of pooled libraries onto the NextSeq 550Dx sequencer (San Diego, CA, USA).

### 2.6. Data Processing and Variant Classification

Raw sequencing reads containing molecular barcodes (Unique Molecular Identifiers, UMIs) were processed using a custom bioinformatic pipeline. Adapter sequences were trimmed using fastp (v0.24.0), followed by alignment to the human reference genome GRCh38 using BWA-mem2 [22,23].

UMI-based error correction was performed using fgbio (v2.4.0; http://fulcrumgenomics.github.io/fgbio/, accessed on 12 January 2026), including read grouping by UMI families and generation of duplex consensus sequences to suppress sequencing errors and PCR artifacts. Somatic variants were called using Mutect2 as implemented in GATK (v4.6.2.0), with variant annotation performed using Ensembl VEP (v111). Gene fusions were detected using FACTERA. Variants were filtered to retain those with allele frequencies ≥5% in targeted panel regions [24].

### 2.7. Statistical Analysis

All statistical investigations were conducted using Graph-Pad PRISM 9 (San Diego, CA, USA), R v4.5.2 (Boston, MA, USA) and SPSS 26 (IBM, Armonk, NY, USA). Baseline clinical characteristics and the frequencies of genomic alterations were compared using a two-sided Fisher’s exact test. Continuous variables were compared using the non-parametric tests. For exploratory enrichment analyses linking genomic alterations with immune marker expression patterns, a nominal significance threshold of *p* < 0.1 was applied to identify candidate associations. Given the exploratory nature of these analyses and the limited cohort size, results were interpreted as hypothesis-generating rather than definitive statistical evidence. Survival measures were analyzed utilizing the Kaplan–Meier method. For statistical comparisons between tumor entities, subgroups with fewer than five cases (head and neck, urological, and skin tumors) were excluded to ensure appropriate group sizes for statistical testing. In contrast, all samples were included in descriptive analyses to provide a comprehensive overview of the molecular and immunological characteristics across the cohort. Additionally, BioRender (Toronto, ON, Canada) was used as a tool for visualization.

## 3. Results

### 3.1. Cohort and Subgroups

Among the 49 patients with BMs, 22 were female (44.9%). The median age at BM-onset was 63.1 years (range 28–84 years). At the time of BM diagnosis, 19 patients (38.8%) exhibited a significantly reduced general condition, indicated by an Eastern Cooperative Oncology Group Performance Status (ECOG-PS) score of ≥2. BMs occurred metachronously in 32 patients (68.1%) and synchronously in 15 patients (31.9%). The median follow-up for the entire cohort was 27.7 months. The overall mortality rate was 57.1% (28 out of 49 cases). Baseline clinical characteristics of the cohort are summarized in Table 1.

### 3.2. Genomic Landscape of Brain Metastases Across Tumor Entities

**Figure 1:**
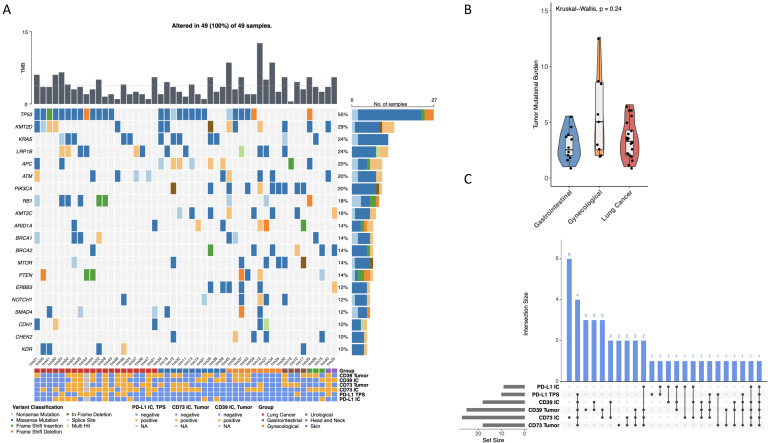
Integrated genomic and immune marker landscape of brain metastases across tumor entities. (**A**) Oncoplot summarizing the mutational landscape of *n* = 49 brain metastases analyzed by targeted panel sequencing. The upper bar plot depicts tumor mutational burden (TMB) per sample. The heatmap displays recurrent somatic alterations in selected genes, with mutation types color-coded. Bar plots on the right indicate overall alteration frequencies; the horizontal dotted line in the upper panel denotes the mean TMB. Clinical annotation tracks below the oncoplot show tumor entity and compartment-specific immunohistochemical marker status, including CD39 and CD73 expression on tumor cells and immune cells (IC), as well as PD-L1 expression assessed as tumor proportion score (TPS) and immune cell (IC) staining. (**B**) Comparison of TMB across the three major tumor entity groups (gastrointestinal, gynecological, lung cancer), demonstrating no significant differences (Kruskal–Wallis test, *p* = 0.24). (**C**) UpSet plot illustrating intersections of CD39/CD73 and PD-L1 expression patterns across tumor and immune compartments, highlighting heterogeneous and only partially overlapping immune phenotypes among brain metastases.

A total of 49 brain metastases derived from multiple primary tumor entities were successfully profiled by targeted panel sequencing. All samples harbored at least one somatic alteration, with a heterogeneous mutational landscape across cases and tumor origins. Frequently altered genes included *TP53* (55%), *KMT2D* (29%), *KRAS* (24%), *LRP1B* (24%), *APC* (20%), *ATM* (20%), and *PIK3CA* (20%), reflecting the genomic diversity typical of advanced metastatic disease. These findings indicate that brain metastases harbor recurrent molecular alterations while maintaining substantial inter-patient heterogeneity (Figure 1A).

TMB varied substantially between samples, without clear clustering by primary tumor entity. To assess whether the immunogenomic landscape might be confounded by differences in mutational load, TMB was compared between the three largest tumor entity groups. TMB values showed considerable variability within each group; however, no statistically significant differences were observed between gastrointestinal, gynecological, and lung cancer metastases (Kruskal–Wallis test, *p* = 0.24). These data suggest that differences in immune marker expression patterns across the cohort are unlikely to be primarily driven by overall mutational burden, supporting the concept that alternative immune escape mechanisms beyond neoantigen load may be relevant in brain metastases.

### 3.3. Heterogeneous Expression of Adenosine Pathway Markers and PD-L1 in Tumor and Immune Compartments

Expression of the adenosine pathway markers CD39 and CD73, as well as PD-L1, was assessed separately in tumor cells and immune cells (IC) (Supplementary Table S4). Across the cohort, expression patterns were highly heterogeneous and only partially overlapping, highlighting substantial diversity of immunoregulatory phenotypes in brain metastases (Supplementary Figure S2). An intersection analysis demonstrated that co-expression of multiple markers occurred in only a subset of cases, whereas many metastases displayed distinct or isolated expression profiles (Figure 1C). Notably, a considerable proportion of tumors exhibited CD39 and/or CD73 positivity in the absence of PD-L1 expression, supporting the presence of a PD-L1–independent immunosuppressive axis in brain metastases. Conversely, PD-L1 positivity (either TPS or IC) was also observed in tumors lacking adenosine pathway activation, indicating that these immune escape programs can occur independently (Supplementary Figure S3). Together, these findings suggest that stratification based solely on PD-L1 status is insufficient to capture the immunosuppressive landscape of brain metastases, and that CD39/CD73 provide additional biologically relevant information.

### 3.4. Genetic Correlation of Compartment-Specific CD39/CD73 Expression and PD-L1 Immune Cell Positivity

**Figure 2:**
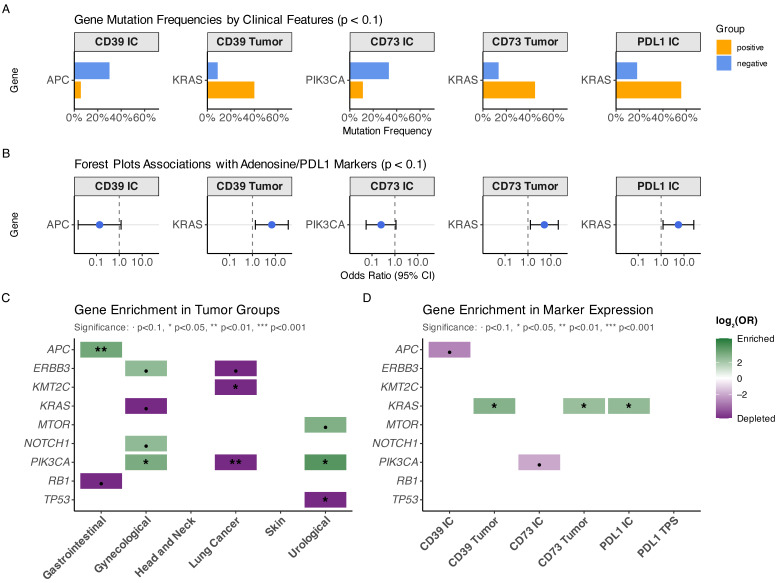
Genetic correlates of adenosine pathway marker and PD-L1 expression in brain metastases. (**A**) Bar plots showing mutation frequencies of selected genes stratified by compartment-specific immunohistochemical marker status, including CD39 expression on immune cells (CD39 IC) and tumor cells (CD39 Tumor), CD73 expression on immune cells (CD73 IC) and tumor cells (CD73 Tumor), and PD-L1 expression on immune cells (PD-L1 IC). Only associations meeting the exploratory significance threshold (*p* < 0.1) are shown. (**B**) Forest plots summarizing the corresponding associations between gene alterations and marker positivity, displayed as odds ratios (OR) with 95% confidence intervals. (**C**) Heatmap illustrating enrichment or depletion of recurrent gene alterations across tumor entity groups (gastrointestinal, gynecological, head and neck, lung cancer, skin, urological), based on log2-transformed odds ratios. (**D**) Heatmap depicting enrichment patterns of gene alterations across immune marker expression subgroups (CD39/CD73 tumor and immune cell expression, PD-L1 IC and TPS). Color intensity reflects log2(OR), with statistical significance indicated by asterisks.

To investigate whether specific genomic alterations are associated with adenosine pathway activation or PD-L1 expression, mutation frequencies were compared between marker-positive and marker-negative subgroups (threshold *p* < 0.1). This analysis identified several associations linking tumor-intrinsic genetics with immune phenotypes (Figure 2A; Supplementary Table S5). Most notably, *KRAS* alterations were enriched in metastases exhibiting tumor cell expression of CD39 as well as tumor cell expression of CD73, suggesting a link between oncogenic RAS signaling and tumor-intrinsic adenosine pathway activation. In addition, *KRAS* mutations were also associated with PD-L1 expression on immune cells, indicating that KRAS-driven tumors may promote both adenosine-mediated and immune cell–adaptive immunoregulatory programs. In contrast, expression of CD39 on immune cells was associated with differences in *APC* mutation status, whereas CD73 immune cell positivity was associated with *PIK3CA* alterations, supporting the concept that immune compartment adenosine signaling is shaped by distinct genetic contexts compared to tumor cell–intrinsic expression. These associations were further supported by odds ratio analyses (Figure 2B), which confirmed enrichment of *KRAS* alterations in CD39 tumor-positive and CD73 tumor-positive metastases, while *APC* and *PIK3CA* showed differential enrichment patterns in immune cell marker expression. Collectively, these results suggest that adenosine pathway activation in brain metastases is not random but may be at least partially genetically wired and compartment-specific.

### 3.5. Tumor Entity–Specific Enrichment of Driver Alterations

Given the multi-entity design of the cohort, explorative enrichment analyses (*p* < 0.1) were performed to determine whether specific genetic alterations were preferentially associated with particular tumor origins. Significant entity-specific enrichment patterns were observed (Figure 2C).

*APC* alterations were strongly enriched in gastrointestinal metastases, consistent with canonical gastrointestinal oncogenic programs [25]. In contrast, *PIK3CA* alterations were enriched in gynecological tumors and lung cancer metastases, underlining that PI3K signaling may represent a recurrent pathway in these subgroups [26]. Additional entity-specific patterns included enrichment of *NOTCH1* and *ERBB3* in selected groups, whereas *TP53* alterations showed relative depletion in the urological subgroup [27]. These findings underscore that while brain metastases share overlapping genomic hallmarks, tumor entity–specific mutational programs persist and may contribute to shaping distinct immunoregulatory environments.

To directly connect tumor genetics with immune marker expression patterns, a marker-centered enrichment analysis was performed across the entire cohort (Figure 2D). This analysis confirmed that *KRAS* alterations were significantly enriched in tumors expressing CD39 and CD73 on tumor cells, and also associated with PD-L1 immune cell positivity.

Conversely, *APC* alterations were depleted in CD39 immune cell–positive metastases, whereas *PIK3CA* alterations showed differential enrichment patterns in immune marker subgroups, suggesting that the genetic background may influence whether immune escape is preferentially mediated through tumor-intrinsic adenosine signaling versus immune cell–driven mechanisms. Together, these data provide evidence that CD39/CD73 expression is not merely an epiphenomenon but reflects distinct molecular contexts in brain metastases.

### 3.6. Recurrent Gene Fusions Are Not Associated with Adenosine/PD-L1 Immuno-Subtypes

**Figure 3:**
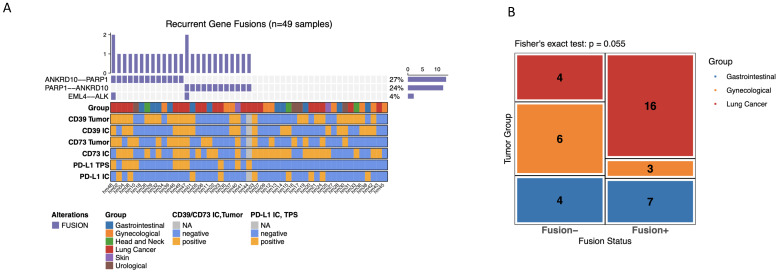
Recurrent gene fusions in brain metastases and their distribution across tumor entities. (**A**) Oncoplot summarizing recurrent gene fusions detected in *n* = 49 brain metastases. The upper bar plot indicates the number of fusion events per sample. The heatmap displays fusion occurrences for the most frequent rearrangements, including *ANKRD10–PARP1*, *PARP1–ANKRD10*, and *EML4–ALK*, with corresponding overall frequencies shown on the right. Annotation tracks below indicate tumor entity as well as compartment-specific immunohistochemical marker status for CD39 and CD73 (tumor cells and immune cells) and PD-L1 (tumor proportion score [TPS] and immune cell [IC] expression). (**B**) Mosaic plot depicting fusion status (fusion-positive vs. fusion-negative) across the major tumor entity groups. Numbers indicate absolute sample counts per group. Fisher’s exact test demonstrates a trend toward unequal distribution of fusion-positive cases across entities (*p* = 0.055).

In addition to point mutations and small indels, recurrent gene fusions were assessed across the cohort (Figure 3A; Supplementary Table S6). Recurrent fusions were selected for visualization based on their frequency within the cohort rather than a priori assumptions. ANKRD10–PARP1/PARP1–ANKRD10 represented the most frequently observed rearrangements and were therefore highlighted, given the involvement of PARP1 in DNA damage repair pathways. EML4–ALK, a canonical driver fusion typically observed in lung adenocarcinoma, was included to provide biological and clinical context. Thus, the displayed fusions reflect recurrence and potential relevance rather than arbitrary selection. Fusion-positive cases were detected in all major tumor entity groups, with a trend toward an unequal distribution between gastrointestinal, gynecological, and lung cancer metastases (Fisher’s exact test, *p* = 0.055; Figure 3B). Lung cancer metastases accounted for the largest absolute number of fusion-positive samples. However, fusion events were also observed in gastrointestinal and gynecological tumors.

Although some fusion-positive cases appeared to show elevated immune marker expression on visual inspection, no statistically significant association between fusion status and adenosine/PD-L1 immune phenotypes was detected. Fusion-positive and fusion-negative metastases were distributed across the full spectrum of marker expression patterns, suggesting that gene fusions represent an additional entity-specific genomic feature of brain metastases that is largely independent of adenosine pathway activation or PD-L1–related immune phenotypes.

### 3.7. Immunogenomic Stratification Reveals Distinct Adenosine- and PD-L1-Driven Subtypes

**Figure 4:**
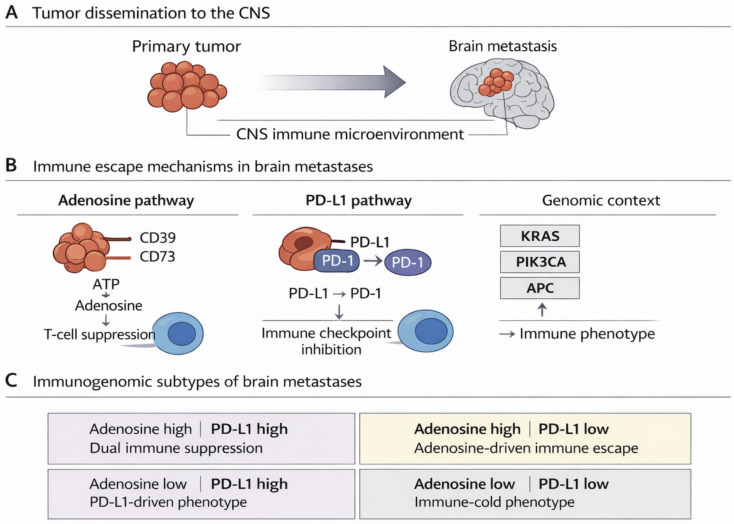
**Conceptual overview of immunogenomic immune escape mechanisms in brain metastases.** (**A**) Schematic illustration of tumor dissemination from the primary tumor to the central nervous system (CNS), where metastatic tumor cells establish within a distinct CNS immune microenvironment. (**B**) Major immune escape mechanisms identified in brain metastases. Tumor cells may suppress anti-tumor immunity through activation of the adenosine pathway via CD39 and CD73, leading to extracellular ATP degradation and adenosine-mediated T-cell inhibition. In parallel, immune checkpoint signaling through PD-L1 expression on tumor cells and PD-1 on T cells contributes to immune suppression. In addition, specific oncogenic alterations, including KRAS, PIK3CA, and APC, are associated with distinct immune phenotypes and may influence the activation of immunoregulatory pathways. (**C**) Integration of adenosine pathway activity and PD-L1 expression defines four immunogenomic subtypes of brain metastases: adenosine-high/PD-L1-high (dual immune suppression), adenosine-high/PD-L1-low (adenosine-driven immune escape), adenosine-low/PD-L1-high (PD-L1–driven phenotype), and adenosine-low/PD-L1-low (immune-cold phenotype). Created with BioRender.com.

To integrate marker expression profiles with tumor entity and genomic alterations, tumors were classified into four immunogenomic subtypes based on combined adenosine pathway activation and PD-L1 status: adenosine high/PD-L1 high, adenosine high/PD-L1 low, adenosine low/PD-L1 high, and adenosine low/PD-L1 low. A Sankey-based integrative visualization demonstrated that these immune subtypes were distributed across tumor entities but exhibited preferential enrichment of specific mutational patterns (Supplementary Figure S4). In particular, KRAS alterations were strongly linked to adenosine-high phenotypes, consistent with the association observed in the marker-centered enrichment analyses. In contrast, other alterations such as *PIK3CA*, *LRP1B*, and *TP53* were distributed across multiple immune subtypes, indicating broader roles in metastatic progression rather than exclusive immune phenotype specification. Importantly, a substantial fraction of cases fell into the adenosine high/PD-L1 low subtype, highlighting a clinically relevant subgroup of brain metastases that may escape immune surveillance through adenosine-mediated suppression despite low PD-L1 expression. This finding supports the concept that targeting the CD39/CD73 adenosine axis may represent a promising strategy to expand immunotherapeutic options beyond classical PD-1/PD-L1 checkpoint inhibition in brain metastases. Neither the underlying histological tumor entity nor the immunogenomic subtype classification showed a significant association with PFS or OS in this cohort in the absence of immune checkpoint blockade therapy (Supplementary Figure S5). Notably, a numerical trend towards inferior outcomes was observed in highly immunogenic brain metastases characterized by combined adenosine pathway activation and high PD-L1 expression (adenosine high/PD-L1 high), which may indicate a clinically relevant high-risk phenotype and further underscore the unmet medical need for an expansion of immunotherapeutic strategies in brain metastases. It should be explicitly stated that the analyses performed were not powered for survival endpoints (Figure 4).

## 4. Discussion

Brain metastases represent a major clinical challenge, in part due to their highly immunosuppressive microenvironment and the frequently limited efficacy of immune checkpoint inhibition (ICI) [7,28]. While PD-1/PD-L1 blockade can induce durable responses in selected extracranial settings, clinical activity within the central nervous system remains heterogeneous, suggesting that additional, brain-specific immune escape programs may restrict therapeutic benefit. In this context, recent work has emphasized metabolic immunosuppression, particularly mediated through the extracellular ATP–adenosine axis, as a critical and potentially targetable mechanism of immune regulation that complements classical checkpoint pathways [29,30]. Consistent with this emerging concept, we found that the adenosine pathway markers CD39 and CD73 are commonly expressed in brain metastases, exhibit pronounced inter-tumoral heterogeneity, and show only partial overlap with PD-L1 expression.

Notably, CD39 and CD73 expression was detectable both within malignant cells and in tumor-infiltrating immune cells, indicating that adenosine signaling operates in a compartment-specific manner. A substantial subset of metastases displayed strong CD39/CD73 positivity despite absent or low PD-L1 expression, supporting the existence of PD-L1-independent immunosuppressive phenotypes in the brain metastatic niche [10]. Similar patterns have recently been reported in extracranial tumors with limited responsiveness to PD-1/PD-L1 blockade, reinforcing the concept that adenosine-mediated immune suppression may represent a convergent resistance mechanism across metastatic contexts [31,32].

Mechanistically, the CD39/CD73 cascade catalyzes the conversion of extracellular ATP released during cellular stress and tissue damage into immunosuppressive adenosine, thereby promoting T-cell dysfunction and myeloid tolerance [33]. Importantly, our data suggest that activation of this pathway in brain metastases is not merely stochastic but is linked to defined tumor-intrinsic genetic programs. In particular, *KRAS* alterations were enriched in tumors exhibiting CD39 and CD73 expression within the malignant compartment, aligning with broader evidence that oncogenic RAS signaling can drive metabolic rewiring and foster immunosuppressive microenvironments. This resonates with immunogenomic frameworks that link tumor-intrinsic alterations to immune modulation [29]. In contrast, *APC*- and *PIK3CA*-associated alteration patterns correlated more strongly with immune cell marker expression, suggesting that distinct oncogenic backgrounds may differentially shape tumor-intrinsic versus microenvironment-driven adenosine signaling [34].

Interestingly, tumor mutational burden did not differ significantly across major tumor entity groups, arguing against mutational load as the primary determinant of the observed immune phenotypes [35]. Instead, these findings support the view that immune escape in brain metastases may be dominated by microenvironmental and metabolic suppression programs that can operate independently of neoantigen burden, consistent with current perspectives questioning the predictive value of TMB in immunologically constrained metastatic niches [35,36].

By integrating genomic profiles with compartment-resolved immune marker expression, we defined four immunogenomic subtypes of brain metastases characterized by distinct combinations of adenosine pathway activation and PD-L1 status. Of particular interest, an “adenosine-high/PD-L1-low” subgroup emerged as a potentially actionable phenotype that may not be adequately captured by conventional PD-L1-based stratification strategies, yet could represent a rational target population for emerging adenosine-directed immunotherapies [33]. This framework extends current immunogenomic concepts into the underexplored setting of brain metastases and provides a clinically interpretable model for biomarker-driven therapeutic approaches [7].

Although recurrent gene fusions were detected in a subset of tumors, fusion status did not show a meaningful association with the defined immune subtypes, indicating that fusion-driven oncogenic events may contribute to genomic heterogeneity without directly shaping the dominant adenosine- or PD-L1-driven immune escape programs identified here.

Some limitations should be considered. Although this cohort represents a comparatively well-characterized multi-entity collection of brain metastases, sample size remains limited for certain tumor subgroups, and thus some enrichment signals should be interpreted as hypothesis-generating rather than definitive associations. Due to the limited cohort size and the relatively small number of events within several molecular subgroups, multivariate regression analyses were not performed to avoid unstable model estimates. In particular, the observed links between genomic alterations and immune marker expression patterns should be regarded as exploratory observations that require validation in larger independent cohorts. Additionally, no sufficient tissue material from the primary tumor was available for analysis; therefore, no correlations could be performed. Consequently, it remains unclear whether the observed immune phenotypes represent intrinsic properties of the primary tumors or reflect adaptive changes occurring during metastatic colonization of the brain microenvironment. However, the primary aim of this study was to characterize the immunogenomic landscape within the CNS metastatic niche, which represents a biologically distinct microenvironment with a unique immune composition compared with peripheral tissues. In many cases included in this cohort, the diagnosis of metastatic disease was established directly from neurosurgically obtained brain metastasis tissue, while archival primary tumor material was not available or was insufficient for molecular analysis. The retrospective cross-sectional design did not allow systematic correlation with immunotherapy response or longitudinal immune evolution. Furthermore, immunohistochemistry provides clinically applicable, compartment-resolved information but remains semi-quantitative and does not directly measure functional adenosine activity. Finally, panel sequencing enables robust detection of recurrent driver alterations, yet cannot fully capture the entire spectrum of genomic complexity. Therefore, TMB estimates should be regarded as relative measures.

## 5. Conclusions

In summary, our study highlights pronounced immunological heterogeneity in brain metastases and demonstrates that PD-L1 expression alone is insufficient to capture the relevant immune escape landscape. Adenosine pathway activation, reflected by compartment-specific CD39 and CD73 expression, is frequent, genetically associated with distinct oncogenic backgrounds including *KRAS*, *APC*, and *PIK3CA*, and enables the definition of clinically interpretable immunogenomic subtypes. These results support the concept that adenosine-mediated immune suppression represents a genetically wired and potentially targetable resistance program in brain metastases, providing a rationale for innovative combination immunotherapy strategies beyond classical checkpoint inhibition.

## Figures and Tables

**Table 1:** Baseline characteristics of the patients included in this study when diagnosed with cerebral metastasis.

Characteristics	Study Cohort (*n* = 49)	NSCLC (*n* = 20)	GI (*n* = 11)	Gynaecologic (*n* = 9)	Head & Neck (*n* = 3)	Urological (*n* = 4)	Skin (*n* = 2)
**Male**	27 (55.1%)	12 (60.0%)	8 (72.7%)	0 (0.0%)	3 (100.0%)	3 (75.0%)	1 (50.0%)
**Female**	22 (44.9%)	8 (40.0%)	3 (27.3%)	9 (100.0%)	0 (0.0%)	1 (25.0%)	1 (50.0%)
**Median age (range, years)**	63.1 (28–84)	62.1 (44–84)	61.8 (28–82)	61.4 (38–76)	64.7 (59–69)	65.5 (57–70)	79.5 (76–83)
**ECOG PS** - 0–1 - >2	30 (61.2%) 19 (38.8%)	16 (80.0%) 4 (20.0%)	7 (63.6%) 4 (26.4%)	5 (55.6%) 4 (44.4%)	1 (33.3%) 2 (66.7%)	1 (25.0%) 3 (75.0%)	0 (0.0%) 2 (100.0%)
**B-symptoms** - Yes - No	15 (30.6%) 34 (69.4%)	6 (30.0%) 14 (70.0%)	4 (36.4%) 7 (63.6%)	3 (33.3%) 6 (66.7%)	0 (0.0%) 3 (100.0%)	0 (0.0%) 4 (100.0%)	2 (100.0%) 0 (0.0%)
**Brain met. occurrence** - Synchronous - Metachronous	15 (31.9%) 32 (68.1%)	11 (57.9%) 8 (42.1%)	3 (27.3%) 8 (72.7%)	0 (0.0%) 8 (100.0%)	1 (33.3%) 2 (67.7%)	0 (0.0%) 4 (100.0%)	0 (0.0%) 2 (100.0%)

Abbreviation: ECOG, eastern cooperative oncology group; GI, gastrointestinal cancer; NSCLC, non small cell lung cancer; skin, melanoma.

## Data Availability

The datasets generated and analyzed during the current study are not publicly available. The sequencing data were generated using the institution’s routine diagnostic next-generation sequencing (NGS) pipeline and were processed together with clinical routine samples. As a result, the raw sequencing data are embedded within routine diagnostic datasets and cannot be released separately. Anonymized aggregated data supporting the findings of this study are available from the corresponding author upon reasonable request and with permission of the responsible ethics committee.

## Supplementary Materials

The following supporting information can be downloaded at: https://www.mdpi.com/article/10.3390/cancers18071087/s1. Table S1. Antibodies used in the present study for immunohistochemistry; Table S2. QC summary of targeted sequencing for brain metastasis samples (separate Excel file); Table S3. Somatic variants detected by targeted panel sequencing in brain metastases (separate Excel file); Table S4. Immunohistochemical findings of CD39 and CD73 on tumor and immune cells of cerebral metastasis in the study cohort; Table S5. Pairwise clinical enrichment analysis of immunohistochemical marker subgroups (separate Excel file); Table S6. Summary of gene fusion events detected in brain metastases (separate Excel file); Figure S1. Visualization of the distribution entity assignment of patients with brain metastases using a flow chart; Figure S2. Representative CD39 and CD73 expression in brain metastases. Representative immunohistochemistry (IHC) images showing expression of the adenosine pathway markers CD39 and CD73 in brain metastases from lung and breast cancer. (A) CD39 in lung cancer. (B) CD73 in lung cancer. (C) CD39 in breast cancer. (D) CD73 in breast cancer; Figure S3. Venn diagram of CD39, CD73 and PD-L1 positivity. (A) represents the TPS. (B) represents the IPS in concordance to Schildhaus et al.; Figure S4. Integrated immunogenomic subtypes of brain metastases across tumor entities. Alluvial (Sankey) plot illustrating the relationship between primary tumor entity (left), immunogenomic subtype (center), and recurrent gene alterations (right) in brain metastases. Immunosubtypes were defined based on combined adenosine pathway activity (CD39/CD73) and PD-L1 expression and categorized as Adenosine High/PD-L1 High, Adenosine High/PD-L1 Low, Adenosine Low/PD-L1 High, and Adenosine Low/PD-L1 Low. Flow width represents the number of samples assigned to each connection, highlighting tumor entity–specific distributions and genetic correlates of distinct immune phenotypes; Figure S5. Progression-free and overall survival according to tumor entity and immunogenomic subtype. Progression-free survival (PFS) and overall survival (OS) of patients with brain metastases stratified by histological tumor entity (lung cancer, gynecological, gastrointestinal) (A–B) and by immunogenomic subtypes defined by adenosine pathway activation (CD39 and/or CD73 positivity ≥5%) and PD-L1 tumor proportion score (TPS ≥5%) (C–D). Panels show PFS (A, C) and OS (B, D). No statistically significant differences in survival were observed between tumor entities or immunosubtypes in this cohort. P values indicate overall group comparisons.
