# Supplementary material for: Adenosine Pathway Activation Defines Genetically Linked Immunosuppressive Subtypes in Solid Tumor Brain Metastases
Source: Cancers (Basel). 2026 Mar 26;18(7):1087. doi: 10.3390/cancers18071087 (PMC13072088; doi:10.3390/cancers18071087)
Supplement: Supplementary file 1 [file cancers-18-01087-s001.zip › cancers-4196306 Supplementary Table S1.pdf]

Table S1. Antibodies used in the present study for immunohistochemistry.

Marker	Producer	Clone	Origin
CD39	Sigma-Aldrich	polyclonal	rabbit
CD73	Sigma-Aldrich	polyclonal	rabbit
PD-L1	Roche	SP 263	rabbit
