# Supplementary material for: Adenosine Pathway Activation Defines Genetically Linked Immunosuppressive Subtypes in Solid Tumor Brain Metastases
Source: Cancers (Basel). 2026 Mar 26;18(7):1087. doi: 10.3390/cancers18071087 (PMC13072088; doi:10.3390/cancers18071087)
Supplement: Supplementary file 1 [file cancers-18-01087-s001.zip › Supplementary Figure 1 Flowchart.pdf]

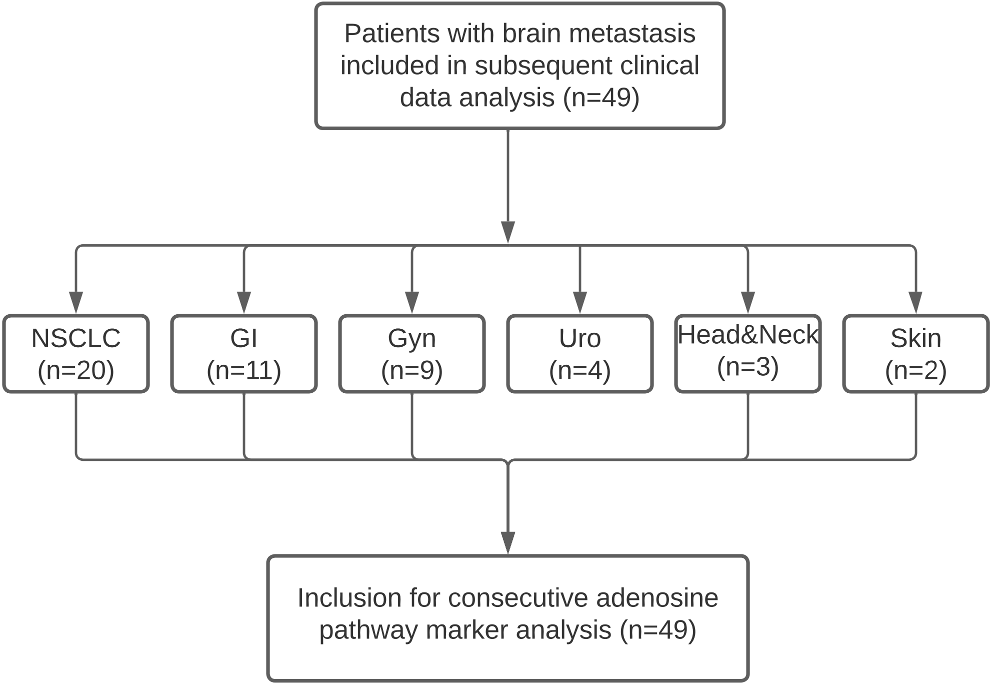
Patients with brain metastasis included in subsequent clinical data analysis (n=49)
NSCLC (n=20)
GI (n=11)
Gyn (n=9)
Uro (n=4)
Head&Neck (n=3)
Skin (n=2)
Inclusion for consecutive adenosine pathway marker analysis (n=49)
