# Supplementary material for: Adenosine Pathway Activation Defines Genetically Linked Immunosuppressive Subtypes in Solid Tumor Brain Metastases
Source: Cancers (Basel). 2026 Mar 26;18(7):1087. doi: 10.3390/cancers18071087 (PMC13072088; doi:10.3390/cancers18071087)
Supplement: Supplementary file 1 [file cancers-18-01087-s001.zip › SupplTable_4- immunohistochemical features NEUdocx.pdf]

Supplementary Table S4: Immunohistochemical findings of CD39 and CD73 on tumor and immune cells of cerebral metastasis in the study cohort

Characteristics	Study cohort (n=49)	NSCLC (n=20)	GI (n=11)	Gynaecologic (n=9)	Head and Neck (n=3)	Urological (n=4)	Skin (n=2)
**Male**	27 (55.1%)	12 (60.0%)	8 (72.7%	0 (0.0%)	3 (100.0%)	3 (75.0%)	1 (50.0%)
**Female**	22 (44.9%)	8 (40.0%)	3 (27.3%)	9 (100.0%)	0 (0.0%)	1 (25.0%)	1 (50.0%)
**TPS CD39 positive**	32 (66.7%)	12 (63.2%)	7 (63.6%)	6 (66.7%)	2 (66.7%)	3 (75.0%)	2 (100.0%)
TPS CD39 (mean, range)	19.7% (0 – 90%)	15.5% (0 – 50%)	24.5% (0 – 80%)	23.4% (0 – 90%)	13.3% (0 – 20%)	8.8% (0 – 15%)	47.5% (5 – 90%)
TPS CD39 <1	16 (33.3%)	7 (36.8%)	4 (36.4%)	3 (33.3%)	1 (33.3%)	1 (25.0%)	0 (0.0%)
TPS CD39 1-5	7 (14.6%)	1 (5.3%)	1 (9.1%)	3 (33.3%)	0 (0.0%)	1 (25.0%)	1 (50.0%)
TPS CD39 >5	25 (52.1%)	11 (57.9%)	6 (54.5%)	3 (33.3%)	2 (66.7%)	2 (50.0%)	1 (50.0%)
**TPS CD73 positive**	24 (50.0%)	14 (73.7%)	4 (36.4%)	2 (22.2%)	0 (0.0%)	3 (75.0%)	1 (50.0%)
TPS CD73 (mean, range)	14.4% (0 – 90%)	18.3% (0 - 80%)	15.5% (0 – 80%)	3.9% (0 – 30%)	0.0% (0 -0%)	12.5% (0 – 30%)	45% (0 - 90%)
TPS CD73 <1	24 (50.0%)	5 (26.3%)	7 (63.6%)	7 (77.8%)	3 (100.0%)	1 (25.0%)	1 (50.0%)
TPS CD73 1-5	6 (12.5%)	5 (26.3%)	0 (0.0%)	1 (11.1%)	0 (0.0%)	0 (0.0%)	0 (0%)
TPS CD73 >5	18 (37.5%)	9 (47.4%)	4 (36.4%)	1 (11.1%)	0 (0.0%)	3 (75.0%)	1 (50.0%)
**IPS CD39 positive**	29 (60.4%)	11 (57.9%)	9 (81.8%)	4 (44.4%)	2 (66.7%)	3 (75.0%)	2 (100.0%)
IPS CD39 (mean, range)	9.1% (0 – 80%)	6.7% (0 – 30%)	7.5% (0 – 20%)	5% (0 – 20%)	28.3% (0 – 80%)	7.5% (0 – 20%)	35% (20 – 50%)
IPS CD39 <1	17 (35.4%)	8 (42.1%)	2 (18.2%)	5 (55.6%)	1 (33%%)	1 (25.0%)	0 (0.0%)
IPS CD39 1-5	13 (27.1%)	4 (22.2%)	5 (45.4%)	1 (11.1%)	1 (33.3%)	2 (50.0%)	0 (0.0%)
IPS CD39 >5	18 (37.5%)	7 (36.8%)	4 (36.4%)	3 (33.3%)	1 (33.3%)	1 (25.0%)	2 (100.0%)
**IPS CD73 positive**	41 (85.4%)	16 (84.2%)	10 (90.9%)	7 (77.8%)	3 (100.0%)	3 (75.0%)	2 (100.0%)
IPS CD73 (mean, range)	15.% (0 – 60%)	18.4% (0 – 60%)	17.7% (0 – 60%)	13.7% (0 – 40%)	13.3% (10 -20%)	3.7% (0 – 5%)	17.5% (15 – 20%)
IPS CD73 <1	7 (14.6%)	3 (15.8%)	1 (9.0%)	2 (22.2%)	0 (0.0%)	1 (25.0%)	0 (0.0%)
IPS CD73 1-5	14 (29.2%)	2 (10.5%)	5 (45.5%)	4 (44.4%)	0 (0.0%)	3 (75.0%)	0 (0.0%)
IPS CD73 >5	27 (56.2%)	14 (73.7%)	5 (45.5%)	3 (33.3%)	3 (100.0%)	0 (0.0%)	2 (100.0%)

Abbreviation: GI, gastrointestinal cancer; IPS, immune proportion score; NSCLC, non-small cell lung cancer; Skin, melanoma; TPS, tumor proportion score.
