# Supplementary figures and images for: Adenosine Pathway Activation Defines Genetically Linked Immunosuppressive Subtypes in Solid Tumor Brain Metastases
Source: Cancers (Basel). 2026 Mar 26;18(7):1087. doi: 10.3390/cancers18071087 (PMC13072088; doi:10.3390/cancers18071087)

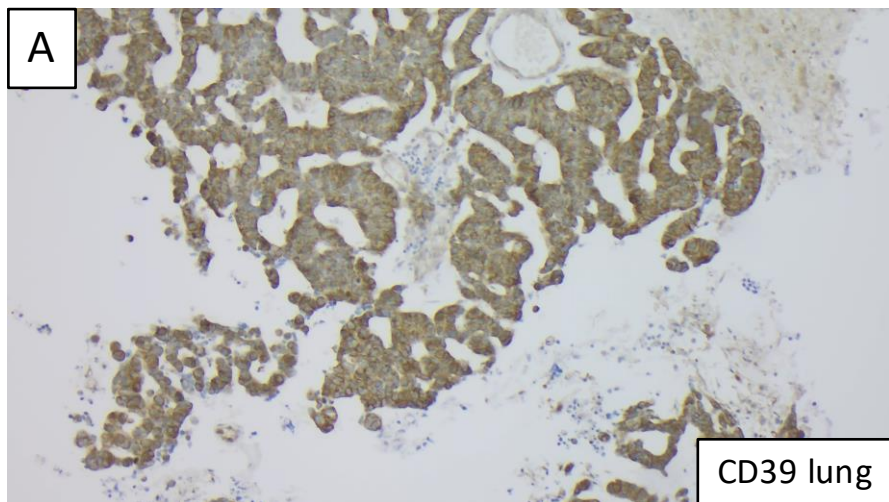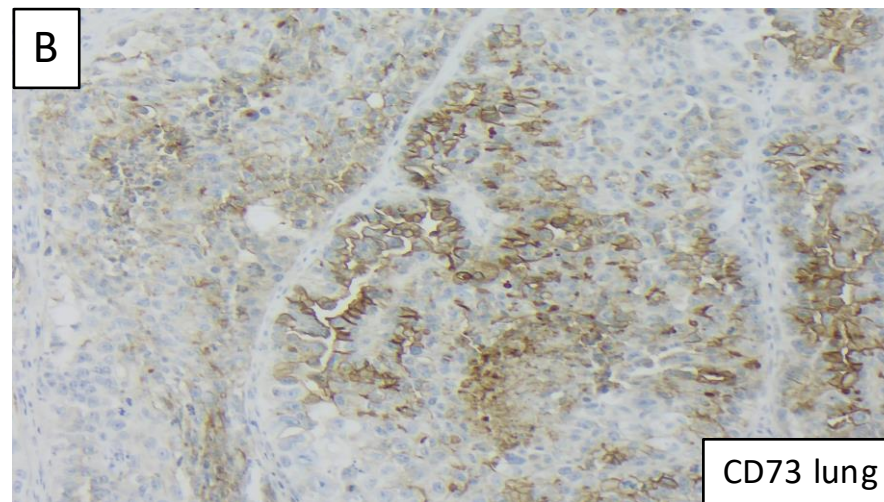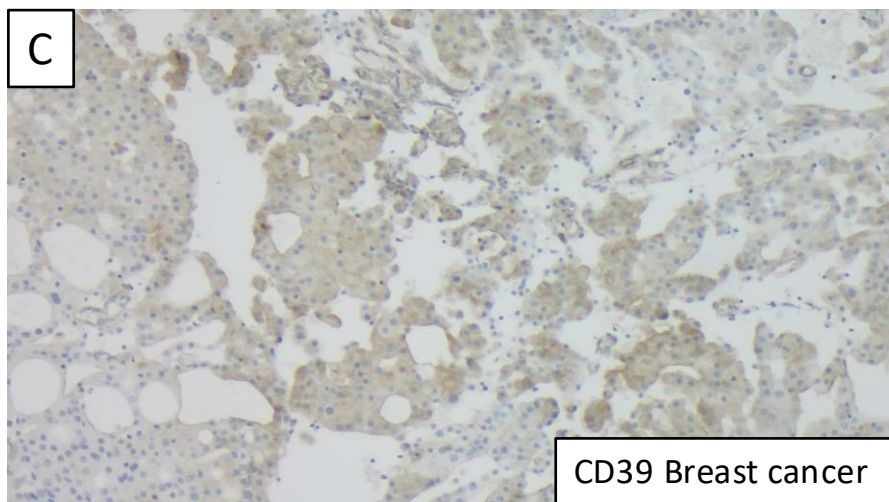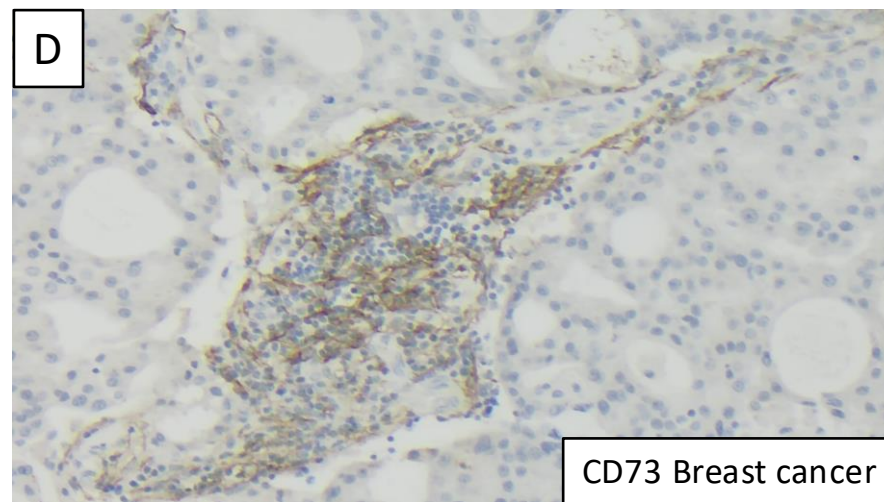

Supplement: Supplementary file 1 [file cancers-18-01087-s001.zip › Supplementary Figure 2 Histology.pdf]

a

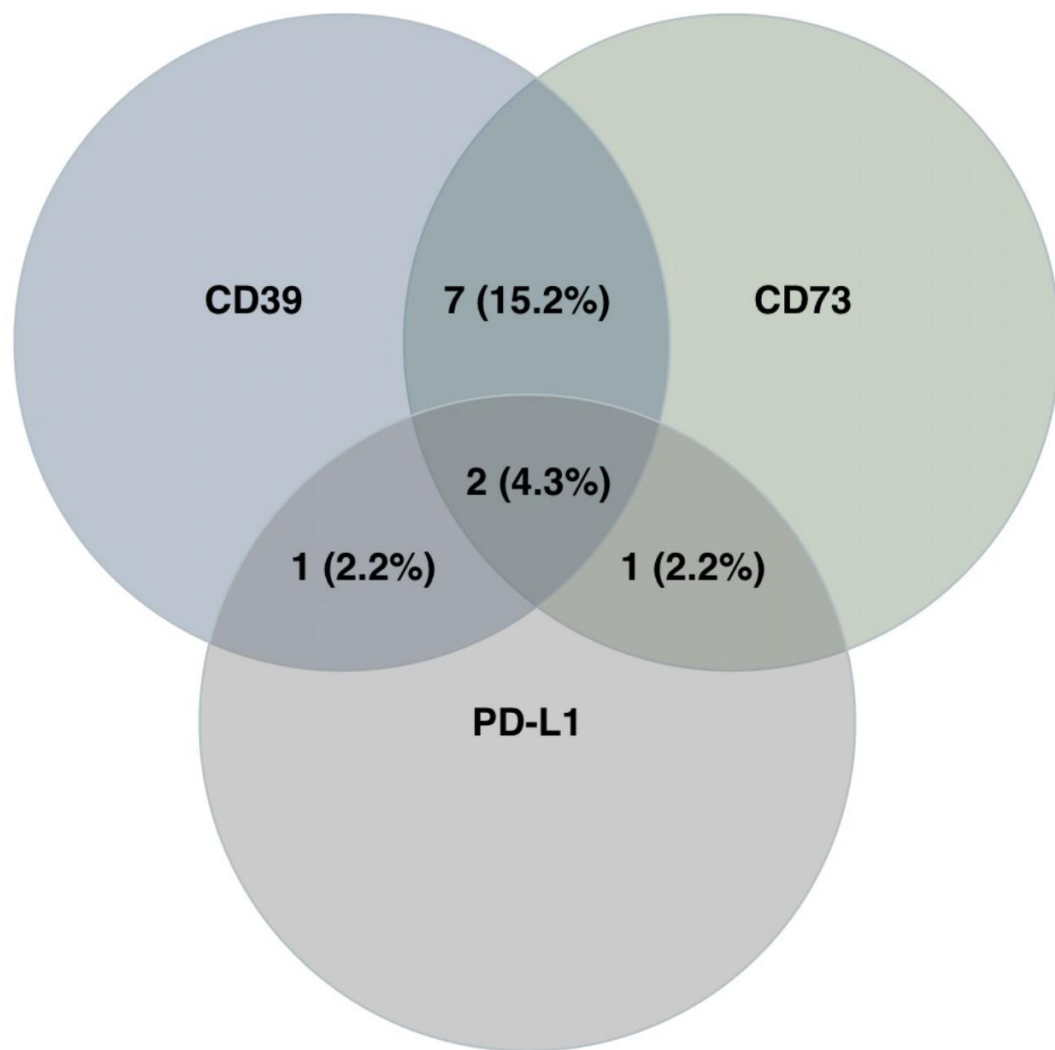

b

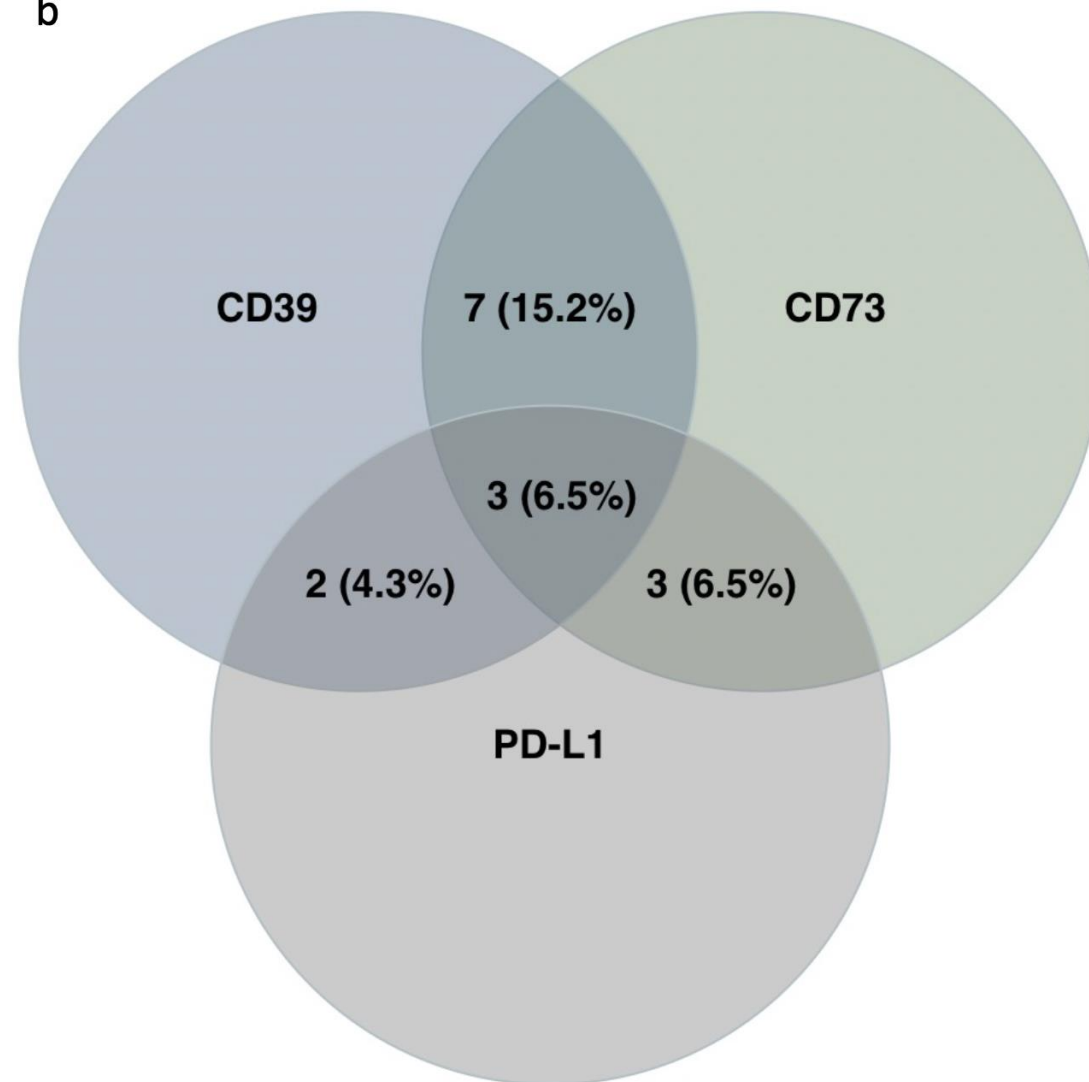

Supplement: Supplementary file 1 [file cancers-18-01087-s001.zip › Supplementary Figure 3 Venn Diagram.pdf]

Immunogenomic Subtypes: Tumor Entity → Immune Phenotype → Mutations

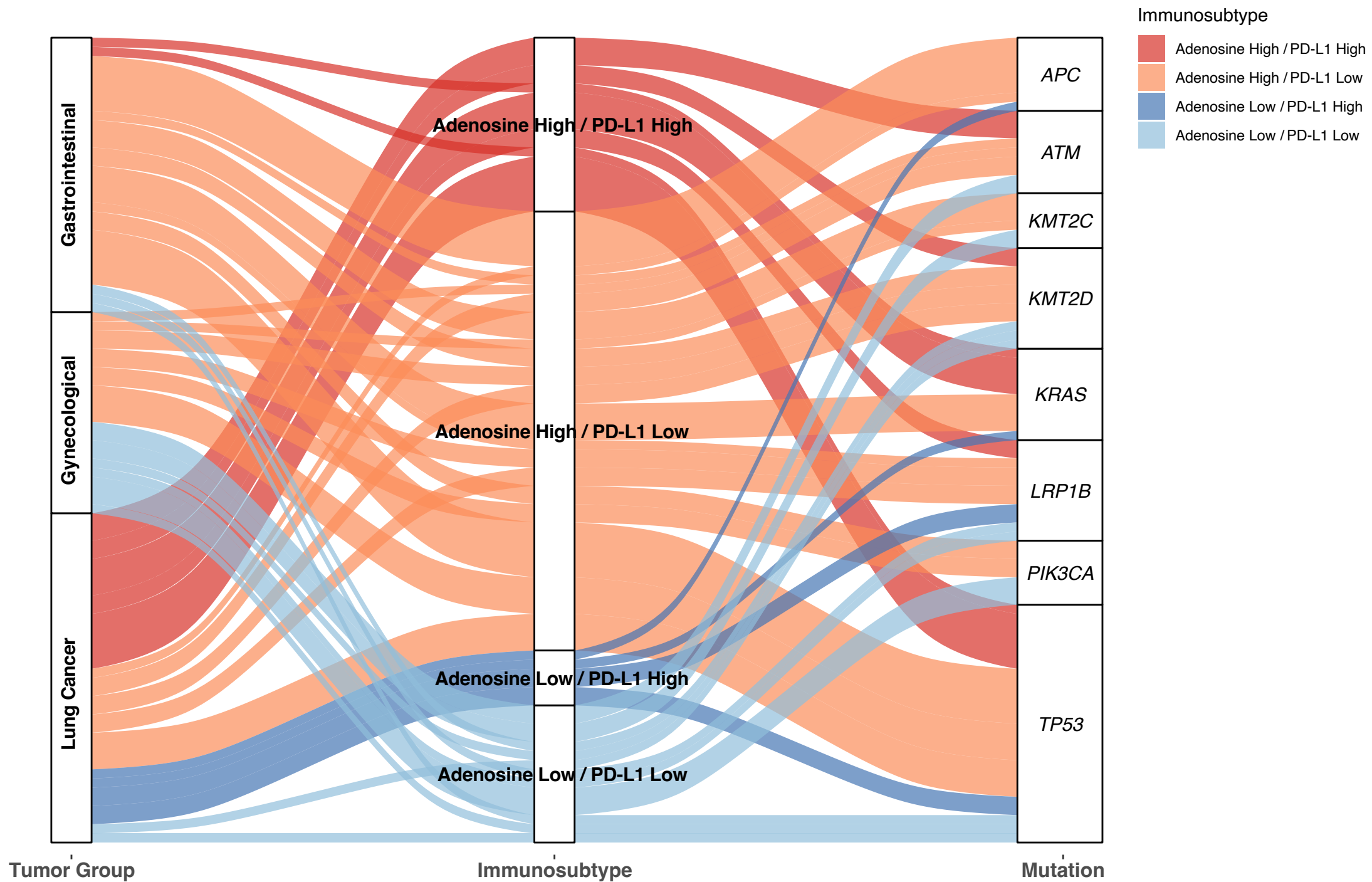

Supplement: Supplementary file 1 [file cancers-18-01087-s001.zip › Supplementary Figure 4 Sankey_Immunosubtypes.EDIT.pdf]

**A**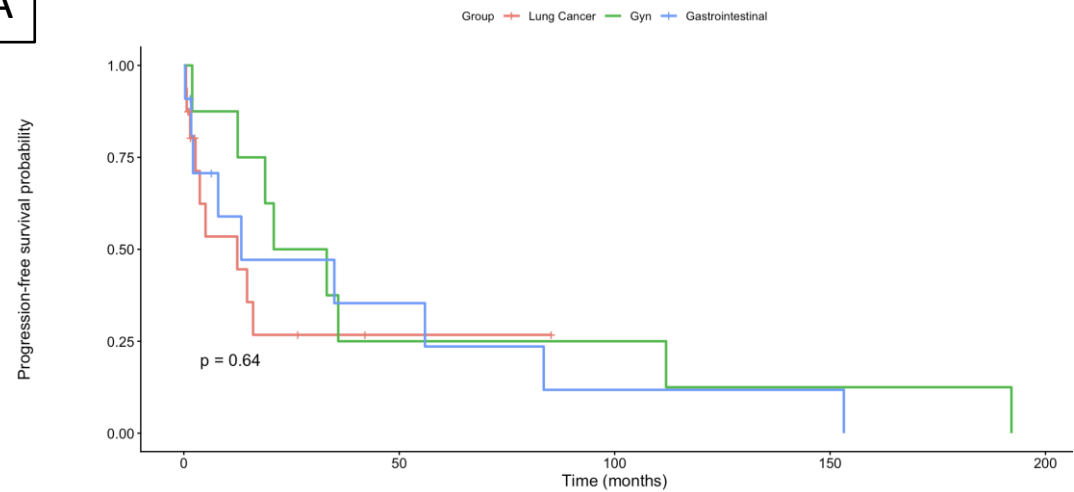**B**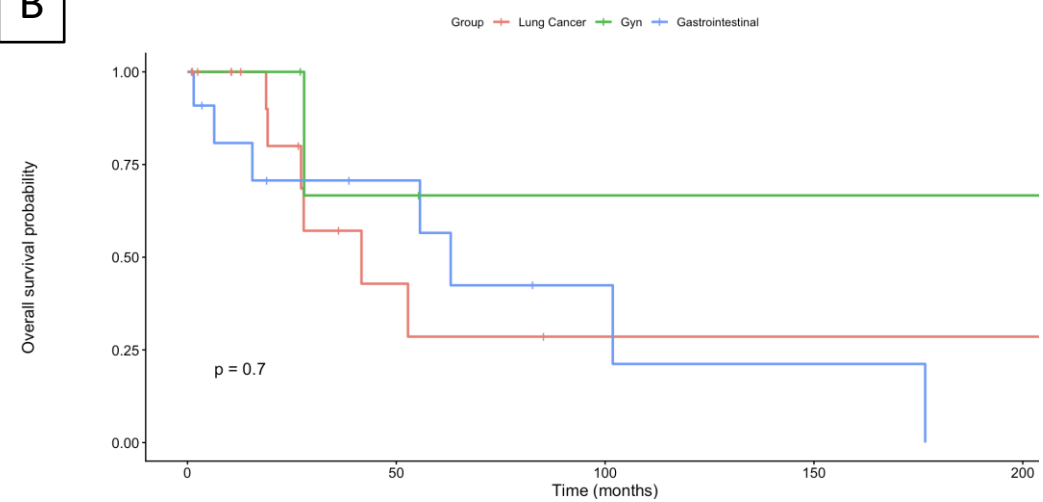**C**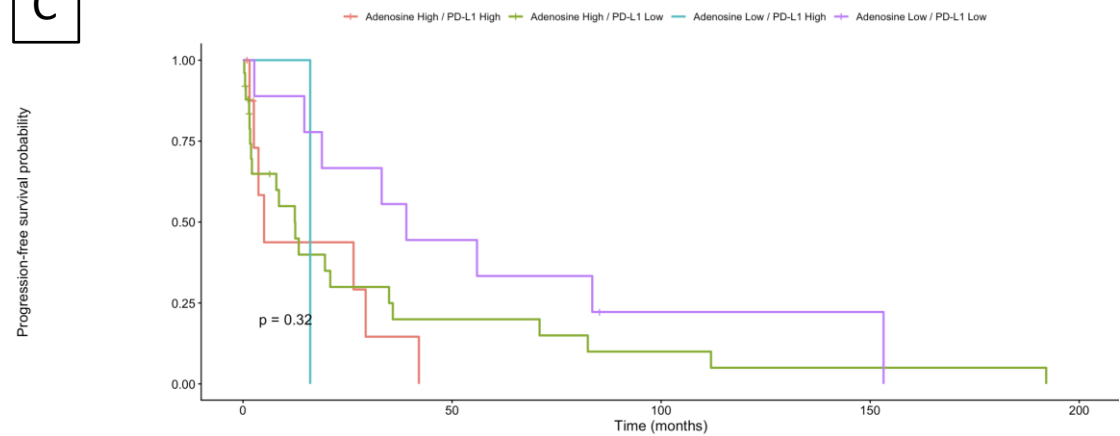**D**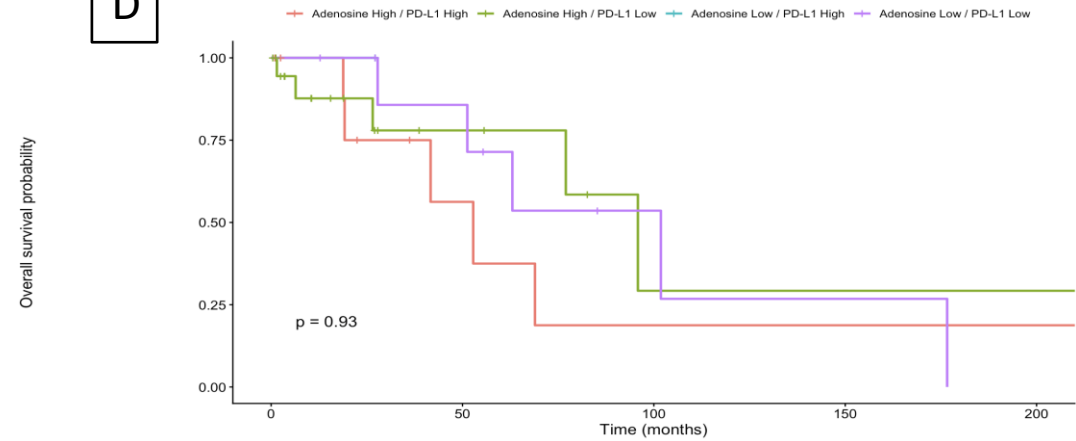

Supplement: Supplementary file 1 [file cancers-18-01087-s001.zip › Supplementary Figure 5.pdf]
